# Sedation efficacy of different dose of remimazolam with sufentanil for nerve block in young and elderly patients: a randomized, controlled study

**DOI:** 10.1007/s00540-022-03142-8

**Published:** 2022-12-03

**Authors:** Xue Li, Tegeleqi Bu, Yu-Ting Li, Ke-Qi Xie, Zhen-Zhen Xu, Xin-Quan Liang, Dong-Liang Mu

**Affiliations:** 1 Department of Anesthesiology, Peking University First Hospital, No. 7 Xishiku Street, Xicheng District, Beijing, 100034 China; 2 Departments of Anesthesiology, Mianyang Central Hospital, Mianyang City, 621000 Sichuan Province China

**Keywords:** Procedure sedation, Remimazolam, Nerve block

## Abstract

**Purpose:**

Anxiety and pain commonly occur during nerve block, we aimed to investigate the sedation efficacy of different doses of remimazolam with sufentanil in young and elderly patients.

**Methods:**

In this randomized trial, patients aged 18–85 years who underwent nerve block was enrolled. All patients received sufentanil 0.08 μg/kg for analgesia. Young patients (age < 65 years) were randomized into the control group (Group C, 0.9% saline), medium-dose remimazolam (Group M, 0.06 mg/kg) and high-dose remimazolam group (Group H, 0.08 mg/kg). Elderly patients (age ≥ 65 years) were randomized into the Group C, low-dose remimazolam group (Group L, 0.04 mg/kg) and Group M. Primary outcome was the success rate of procedure sedation. Respiratory depression and hypoxia were the interested safety outcomes.

**Results:**

Ninety young and 114 elderly patients were enrolled, respectively. In comparison with Groups C and M, young patients in Group H had the highest success rate of procedure sedation (80.0 vs. 73.3 vs. 43.3%, *P* = 0.006). Elderly patients in Groups M and L had similar success rates of procedure sedation, which were significantly higher than that in Group C (78.9 vs. 78.9 vs. 50.0%, *P* = 0.007). In elderly patients, the incidence of respiratory depression and hypoxia tended to be higher in Group M than those in Groups L and C (both *P* < 0.001).

**Conclusion:**

Remimazolam 0.08 mg/kg provided the best sedation efficacy in young patients while remimazolam 0.04 mg/kg with the trend of less respiratory adverse events was more optimal for elderly patients.

**Trial registration:**

http://www.chictr.org.cn/showproj.aspx?proj=122016.

**Supplementary Information:**

The online version contains supplementary material available at 10.1007/s00540-022-03142-8.

## Introduction

Peripheral nerve block presents significant clinical benefits in perioperative pain management [1]. With advanced development in ultrasound technique and its guidance, this invasive procedure can be completed within several minutes. However, any medical intervention embeds with potential risks. The incidences of anxiety and pain are about 60 and 50%, respectively, in patients receiving nerve block at first time [2]. Both of them are considered as major contributors to failure of nerve block, nerve damage, puncture of blood vessels, and visceral organs [2–4].

It is still controversial whether sedation and analgesia should be proposed routinely for nerve block [5]. Procedure sedation is the most common method to alleviate anxiety and pain, during which patient’s consciousness level can purposefully respond to verbal commands or light tactile stimulation [6]. Compared to awake, procedure sedation for nerve block was associated with a 20% reduction of repeated attempts and a 40% reduction of block failure in a large registry analysis including 42, 654 patients [7]. Opposing opinion argues that use of sedatives and analgesics (i.e., midazolam and opioids) increases the risk of over-sedation, hypoxia, nausea and vomiting [7]. Selecting optimal dose of drugs may facilitate to reduce those adverse events [8]. However, potential risk still exists because traditional sedative drugs usually have a relative long-acting time which is much longer than the time needed to perform nerve block, for example, the elimination half-life of midazolam is about 2 h [9].

Remimazolam, an ultrashort-acting benzodiazepine, presents advantage in fast onset, relatively short recovery time with a context-sensitive half time only 6.8 min [10, 11]. Studies showed that remimazolam was associated with superior sedation efficacy to midazolam or propofol in patients undergoing endoscopy [12, 13]. However, there is limited evidence to illustrate its efficacy and safety during nerve block.

Present study was designed to investigate the sedation efficacy of different doses of remimazolam with sufentanil in young and elderly patients who received nerve block, respectively.

## Methods

### Ethics approval

This randomized, double-blind, controlled study conformed to the standards of the Declaration of Helsinki and was approved by the Biomedical Research Ethics Committee of Peking University First Hospital (Ethical Committee no. 2020-426) on 5 January 2021. Written informed consent was obtained from all participants before enrollment. The current study adhered to the CONSORT guidelines and was registered prior to patient enrollment at the Chinese Clinical Trial Registry (http://www.chictr.org.cn/showproj.aspx?proj=122016, ChiCTR2100043530, Date of registration: 21 February, 2021).

### Study design and participants

Patients aged 18–85 years who received nerve block before surgery were enrolled. Present study was conducted to investigate the efficacy of remimazolam for procedure sedation with different regimens in the young (aged < 65 years) and elderly (aged ≥ 65 years) patients separately. The two parts shared the same exclusion criteria: (1) nerve block contraindications including local infection, coagulopathy, and abnormal anatomy; (2) allergy to local anesthetics and benzodiazepines; (3) chronic opioid addiction or use of other analgesics for more than 3 months; (4) inability to communicate due to severe dementia, language barrier or end-stage diseases; or (5) American Society of Anesthesiologists physical status of 4 or above.

### Randomization and masking method

Random numbers were generated for each part using the SAS statistical package version 9.3 (SAS Institute, Cary, NC, USA) in a ratio of 1:1:1 with a block size of 6. Random numbers were enveloped in opaque bags and were allocated to participants in sequence before nerve block.

Trial drugs (either remimazolam or normal saline) were colorless and were prepared with the same brand syringes by a designated nurse (J-M W) who did not take part in drug administration and outcome evaluation. Thus, blind method was kept to patients, researchers in charge of outcome assessment and other healthcare providers.

### Group allocation and intervention

All patients received 0.08 μg/kg sufentnail for prior analgesia. Young patients were randomized to control group (Group C, normal saline), medium-dose (Group M, 0.06 mg/kg) and high-dose (Group H, 0.08 mg/kg) remimazolam for procedure sedation. In elderly patients, the dose of remimazolam was reduced to low-dose (Group L, 0.04 mg/kg) and medium-dose (Group M, 0.06 mg/kg).

### Procedure sedation and data collection

Baseline data including demographic characteristics, comorbidities, and education years were recorded. Subjective sleep quality at the night before surgery was assessed with the numeric rating score (NRS, a 11-point scale where 0 indicating the best sleep and 10 indicating the worst sleep). Preoperative anxiety was assessed by State Trait Anxiety Inventory-6 (STAI-6, ranged from 6 to 24, with higher score indicating heavier anxiety status) [14].

No premedication was given in prior. Patients received standard monitoring including heart rate (HR), non-invasive systolic blood pressure (SBP) and mean arterial pressure (MAP), respiratory rate (RR), pulse oxygen saturation (SpO_2_), and bispectral index (BIS). Sufentnail 0.08 μg/kg was given to all patients via venous access. Two minutes later, trial drugs were administered by attending anesthesiologists. Sedation level was assessed by the modified observer’s assessment of alert/sedation scale (MOAA/S). MOAA/S is a 6-point scale for sedation level assessment (5 for awake, 4 for light sedation, 2–3 for moderate sedation, 0–1 for deep sedation) [15, 16].

Ultrasound-guided nerve block was initiated once MOAA/S score reached to 4 or less. A dose of 0.02 mg/kg remimazolam was added for rescue sedation and could be repeated if necessary. Pain intensity was assessed with the numeric rating score (NRS, a 11-point scale where 0 for no pain and 10 for the worst pain) during the block. If patients complained moderate or severe pain (defined as NRS score ≥ 4), a rescued dose of 0.04 μg/kg sufentanil could be added. NRS of pain was recorded as 0 if patients were in deep sedation (MOAA/S score ≤ 1).

Once SpO_2_ was less than 90%, oxygen was delivered through mask with verbal and tactile stimulation, jaw-thrust and manual-assisted ventilation could be used in necessary.

### Outcomes

Primary outcome was the success rate of procedure sedation, which met with the five criteria simultaneously during the nerve block: (1) MOAA/S score ≤ 4; (2) no unintended body movements which interfered with manipulation of nerve block; (3) no complaint of moderate or severe pain; (4) no requirement for rescue sedation or analgesic medication; and (5) increment of blood pressure and heart rate within 20% baseline value.

Secondary outcomes included the following items: (1) the onset time of sedation (the time interval from initial administration of trial drugs to MOAA/S score ≤ 4); (2) the worst pain score during the block; (3) time consumption of the nerve block (from puncture of skin to withdraw the needle); (4) percentage of using rescued drugs; (5) recovery time (the time interval from last dose of sedatives to MOAA/S score = 5); (6) satisfaction score of sedation from patients and anesthesiologists, assessed with visual analogue scale (VAS, a continuous scale ranged from 0 to 100, where 0 = extremely dissatisfied and 100 = very satisfied) at 30 min after initially administrating trial drugs; (7) BIS, HR, MAP values every 1 min within the first 5 min after administrating trial drugs and then every 5 min thereafter up to 30 min.

Safety outcomes were monitored from administration of trial drugs to 30 min later. Trial-related adverse events included: hypotension (SBP < 90 mmHg), hypertension (SBP > 180 mmHg), bradycardia (HR < 50 beats per minute), tachycardia (HR > 100 beats per minute); respiratory depression (RR < 10 rates per minute), hypoxia (SpO_2_ < 90%), nausea and vomiting.

### Statistical analysis

#### Sample size estimation

In our routine practice, the success rate of procedure sedation was about 40% in young patients and 50% in elderly patients when used sufentanil only. Based on preliminary observation, additional use of 0.06 mg/kg remimazolam in 20 young patients and 0.04 mg/kg in 20 elder patients increased the success rate to 75 and 80%, respectively. Assumed the significance level at 0.05, power at 0.8, and a drop-out rate of 5%, the sample size required to detect difference was 90 in the young patients and 114 in the elderly patients. Sample size calculation was performed with the PASS software (version 15.0, NCSS PASS, Utah, USA).

#### Outcome analysis

Normally distributed continuous variables were compared using analysis of variance (ANOVA) if the homogeneity of variance assumptions was satisfied. Nonnormally distributed continuous variables and ordinal data were compared using the Kruskal–Wallis test. Categorical variables were compared with *χ*^2^ test or Fischer’s exact test. Repeatedly measured variables (BIS, HR, MAP) were compared with the two-way repeated measures ANOVA.

Our primary outcome, the success rate of procedure sedation was compared with *χ*^2^ test, with differences between groups expressed as relative risk and 95% CI. Regarding to pairwise comparation of nonnormally distributed continuous data in secondary outcomes, the median differences and their 95% CI between groups were estimated by Hodges–Lehmann method. Intention- to-treat (ITT) analysis was performed for this study.

A two-sided *P* < 0.05 was considered as statistically significant. The Bonferroni method was used to adjust for multiple comparison. All statistical analyses were performed with the SPSS 26.0 software (IBM SPSS Inc., Chicago, IL, USA).

## Result

### Participants

**Fig. 1:**
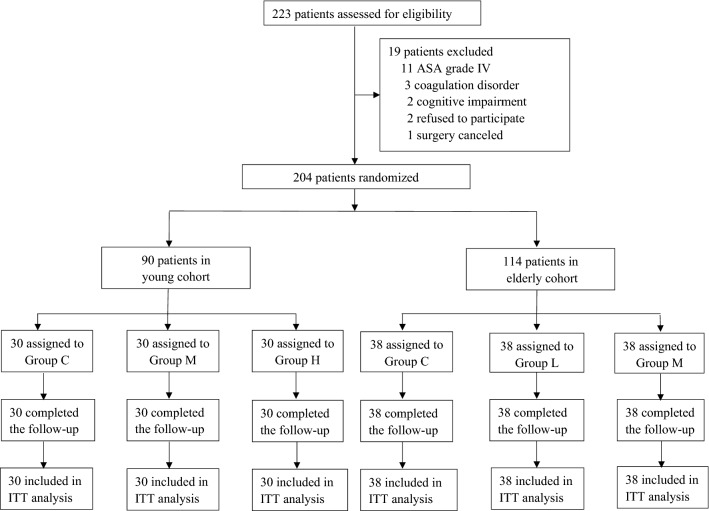
Flow diagram of the study. *ITT* intention-to-treat

From April 6, 2021 to September 1, 2021, a total of 223 patients were screened for eligibility. Ninety young patients (30 in each group) and 114 elderly patients (38 in each group) were randomized, respectively. No patient was lost during follow-up (Fig. 1).

**Fig. 2:**
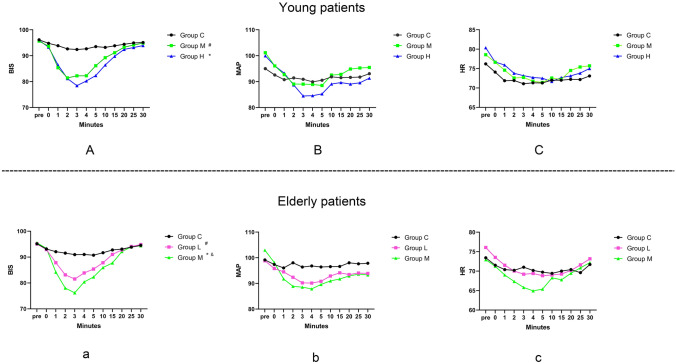
The trend of BIS, MAP and HR among each group (young patients: **A**–**C**; elderly patients: **a**–**c**). Difference between groups were compared by repeated measure of ANOVA. *BIS* bispectral index, *MAP* mean arterial pressure, *HR* heart rate, *ANOVA*: analysis of variation, Time_pre_ indicating the timepoint when patients arrived at the pre-operation room, Time_0_ indicating the timepoint when the trial drugs were administrated. ^#^ *P < 0.0167, compared with Group C; ^&^*P* < 0.0167, compared with Group L

All baseline data in young and elderly patients were balanced among each group except the total dose of remimazolam (Table 1). The trends of BIS, MAP, and HR were depicted in Fig. 2. Repeated measure ANOVA of BIS value showed that, in young and elderly patients, the decrease of BIS value was dose-dependent and the mean BIS value in patients receiving remimazolam were significantly lower than that in Group C (all *P* < 0.05). However, the mean BIS value was only significantly different between Group L and Group M in elderly patients (mean difference = 2.2, 95% CI 0.1–4.3, adjusted *P* = 0.041). No significant difference was found among groups in terms of the mean value of MAP in all patients. The effect of remimazolam on HR seemed minimal except that 0.06 mg/kg remimazolam leaded to an obvious HR decease in the first 5 min in elderly patients.

**Table 1:** Baseline data

Variables	Young patients				Elderly patients			
	Group C (*n* = 30)	Group M (*n* = 30)	Group H (*n* = 30)	*P*	Group C (*n* = 38)	Group L (*n* = 38)	Group M (*n* = 38)	*P*
Age, year	53 (45, 61)	58 (48, 61)	53 (45, 57)	0.256	70 (67, 75)	69 (67, 74)	70 (67, 74)	0.654
Body mass index, kg/m^2^	23.8 (20.8, 25.2)	23.2 (22.1, 24.5)	23.7 (22.1, 25.3)	0.926	23.9 (21.8, 25.0)	24.3 (22.5, 26.9)	23.5 (22.1, 24.3)	0.293
Male, *n*	13 (43.3%)	20 (66.7%)	19 (63.3%)	0.141	22 (57.9%)	22 (57.9%)	30 (78.9%)	0.085
Duration of education, year	12 (10, 16)	12 (9, 15)	15 (12, 16)	0.344	9 (6,15)	12 (9,16)	12 (9,16)	0.225
Comorbidities, *n*								
Stroke	0 (0.0%)	0 (0.0%)	0 (0.0%)	–	4 (10.5%)	2 (5.3%)	5 (13.2%)	0.619
Pulmonary disease	0 (0.0%)	0 (0.0%)	0 (0.0%)	–	2 (5.3%)	2 (5.3%)	1 (2.6%)	> 0.999
Hypertension	6 (20.0%)	12 (40.0%)	8 (26.7%)	0.220	23 (60.5%)	13 (34.2%)	15 (39.5%)	0.051
Chronic heart disease^a^	0 (0.0%)	3 (10.0%)	2 (6.7%)	0.363	7 (18.4%)	6 (15.8%)	7 (18.4%)	0.941
Diabetes mellitus	1 (3.3%)	3 (10.0%)	1 (3.3%)	0.613	6 (15.8%)	4 (10.5%)	6 (15.8%)	0.748
ASA classification, *n*				0.638				0.138
I	10 (33.3%)	8 (26.7%)	7 (23.3%)		3 (7.9%)	4 (10.5%)	4 (10.5%)	
II	16 (53.3%)	17 (56.7%)	21 (70.0%)		19 (50.0%)	28 (73.7%)	25 (65.8%)	
III	4 (13.3%)	5 (16.7%)	2 (6.7%)		16 (42.1%)	6 (15.8%)	9 (23.7%)	
Subjective sleep quality, score^b^	3 (2, 6) *n* = 27	2 (1, 5) *n* = 29	4 (2, 6) *n* = 28	0.613	2 (0, 4) *n* = 37	2 (0, 5) *n* = 37	2 (2, 5) *n* = 35	0.654
State trait inventory-6, score^c^	12.0 ± 2.8	12.0 ± 2.5	11.4 ± 2.4	0.552	10 (9, 13)	10 (9, 12)	11 (9, 12)	0.186
Types of regional block, *n*				0.522				> 0.999
Trunk block^d^	26 (86.7%)	29 (96.7%)	27 (90.0%)		32 (84.2%)	32 (84.2%)	32 (84.2%)	
Extremity block^e^	4 (13.3%)	1 (3.3%)	3 (10.0%)		6 (15.8%)	6 (15.8%)	6 (15.8%)	
Total dose of sufentanil, μg	6.4 (5.0, 7.9)	5.4 (4.9, 6.0)	5.5 (5.0, 6.0)	0.022	5.7 (5.0, 6.0)	5.6 (5.0, 6.0)	5.3 (5.0, 6.0)	0.235
Total dose of remimazolam, mg	0 (0, 0)	4.1 (3.7, 4.5)	5.5 (5.0, 6.0)	< 0.001	0 (0, 0)	2.8 (2.5, 3.2)	4.0 (3.8, 4.2)	< 0.001

Data are presented as mean ± SD, median (interquartile range), or number (%)

*ASA* America Society of Anaesthesiologists

^a^Chronic heart disease included coronary heart disease, arrythmia, valve heart disease

^b^Assessed with the numeric rating score (0 indicates the best sleep quality while 10 equals to the worst sleep quality)

^c^Assessed with the State Trait Anxiety Inventory-6 scale (scores range from 6 to 24, with higher scores indicating heavier anxiety status)

^d^Including transverse abdominis plane block, thoracic paravertebral block, erector spinal plane block, quadratus lumborum, and rectus sheath block; Detailed distribution of each block was listed in Supplement Table S3

^e^Including brachial plexus block, femoral block, and sciatic block; Detailed distribution of each block was listed in Supplement Table S3

### Primary outcome

For young patients in Group H, the success rate of procedure sedation was significantly higher than that in patients who received medium dosage of remimazolam and control group (80.0% in Group H, 73.3% in Group M, and 43.3% in Group C, *P* = 0.006, Table 2). Pairwise comparison showed that statistical difference only existed between Group H and Group C (RR = 1.85, 95% CI 1.18–2.89, *P* = 0.003, Table S1).

**Table 2:** Efficacy outcomes

	Young patients				Elderly patients			
	Group C (*n* = 30)	Group M (*n* = 30)	Group H (*n* = 30)	*P*	Group C (*n* = 38)	Group L (*n* = 38)	Group M (*n* = 38)	*P*
Primary outcome								
Successful rate of sedation, *n*^a^	13 (43.3%)	22 (73.3%)	24 (80.0%)	0.006	19 (50.0%)	30 (78.9%)	30 (78.9%)	0.007
Secondary outcomes								
Onset time, min ^b^	2.0 (2.0, 3.0)	1.0 (1.0, 1.5)	1.0 (1.0, 1.0)	< 0.001	2.0 (2.0, 2.0)	1.0 (1.0, 2.0)	1.0 (1.0, 1.0)	< 0.001
Worst NRS of pain during block, score	3 (2, 5)	1 (0, 3)	1 (0, 3)	< 0.001	2 (0, 4)	1 (0, 2)	0 (0, 2)	0.018
Time consumption of nerve block, min	3.0 (2.5, 4.0)	3.0 (2.0, 5.0)	3.0 (2.0, 5.0)	0.911	3.0 (3.0, 4.0)	3.0 (2.0, 5.0)	4.0 (3.0, 5.0)	0.499
Requirement of rescued drugs, *n*	15 (50.0%)	2 (6.7%)	0 (0.0%)	< 0.001	11 (28.9%)	3 (7.9%)	0 (0.0%)	< 0.001
Recovery time, min^c^	8.0 (5.0, 10.0)	11.0 (8.0, 13.0)	12.0 (10.0, 14.0)	< 0.001	9.0 (6.0, 11.0)	11.0 (7.0, 13.0)	15.0 (10.0, 18.0)	< 0.001
Patient satisfaction, score^d^	80 (80, 90)	100 (90, 100)	100 (100, 100)	< 0.001	90 (80, 100)	100 (100, 100)	100 (100, 100)	< 0.001
Anesthesiologist satisfaction, score^d^	100 (80, 100)	100 (90, 100)	100 (100,100)	0.385	100 (80,100)	100 (100, 100)	100 (80,100)	0.013

Data are median (interquartile range) or number (%)

*NRS* numeric rating score

^a^Defined as meeting with the following five criteria simultaneously: MOAA/S ≤ 4 during nerve block; no unintended body movements which interfered with manipulation of nerve block; no complaint of moderate or severe pain; no requirement for rescue sedation or analgesic medication; increment of blood pressure and heart rate within 20% baseline value

^b^Defined as the time interval from initial administration of trial drugs to MOAA/S score ≤ 4

^c^Defined as the time interval from last dose of sedatives to MOAA/S score = 5

^d^Assessed with visual analogue scale (VAS, a continuous scale ranged from 0 to 100, where 0 = extremely dissatisfied and 100 = very satisfied) at 30 min after initially administered trial drugs

Elderly patients who received either medium or low dose of remimazolam had similar success rate of procedure sedation (78.9% in Group M vs. 78.9% in Group L, *P* > 0.999), both of which were significantly higher than that in Group C (50.0%, both *P* = 0.008, Table 2 and Table S2).

### Secondary outcomes

As Table 2 showed, in combination with any dose of remimazolam, the onset time was shortened by 1 min either in young patients or elderly patients (all *P* < 0.001, Tables S1–2), while the recovery time was prolonged (both *P* < 0.001). Patients who were treated by remimazolam had less pain intensity, less rescued drugs requirement during nerve block, and higher patient satisfaction score in comparison with control group (all *P* < 0.05). Anesthesiologist satisfaction score was similar in each young groups (*P* = 0.385), but it was differed between Group L and control group in elderly patients (*P* = 0.003, Table S2).

### Safety outcome

Regarding to safety outcomes listed in Table 3, patients given remimazolam were more likely to experience respiratory depression (*P* = 0.003, *P* < 0.001) and hypoxia (*P* = 0.043, *P* < 0.001), however, the percentage of jaw-thrust was similar among groups (*P* = 0.770, *P* = 0.772). To be noted, the percentage of patients who were fully aware of the block was less in patients given remimazolam (both *P* < 0.001). The percentage of hypotension, hypertension, as well as nausea and vomiting were similar among groups in all patients.

**Table 3:** Safety outcomes

	Young patients				Elderly patients			
	Group C (*n* = 30)	Group M (*n* = 30)	Group H (*n* = 30)	*P*	Group C (*n* = 38)	Group L (*n* = 38)	Group M (*n* = 38)	*P*
Hypotension^a^	0 (0.0%)	0 (0.0%)	2 (6.7%)	0.326	0 (0.0%)	0 (0.0%)	0 (0.0%)	–
Hypertension^b^	0 (0.0%)	1 (3.3%)	0 (0.0%)	> 0.999	1 (2.6%)	0 (0.0%)	0 (0.0%)	> 0.999
RR < 10 rates/min	0 (0.0%)	5 (16.7%)	9 (30.0%)	0.003	0 (0.0%)	7 (18.4%)	16 (42.1%)	< 0.001
SpO_2_ < 90%	2 (6.7%)	9 (30.0%)	9 (30.0%)	0.043	4 (10.5%)	14 (36.8%)	22 (57.9%)	< 0.001
Jaw thrust maneuver	0 (0.0%)	1 (3.3%)	2 (6.7%)	0.770	1 (2.6%)	0 (0.0%)	2 (5.3%)	0.772
Awareness of block	22 (73.3%)	9 (30.0%)	6 (20.0%)	< 0.001	28 (73.7%)	15 (39.5%)	9 (23.7%)	< 0.001
Nausea and Vomiting	1 (3.3%)	0 (0.0%)	0 (0.0%)	> 0.999	5 (13.2%)	2 (5.3%)	2 (5.3%)	0.502

Data are number (%)

^a^Defined as systolic blood pressure < 90 mmHg

^b^Defined as systolic blood pressure > 180 mmHg

## Discussion

Present study showed that supplementation of remimazolam to sufentanil improved the success rate of procedure sedation both in young and elderly patients during nerve block. These patients also experienced lower pain intensity and higher self-reported satisfaction. To be noted, remimazolam 0.08 mg/kg provided the best sedation efficacy in young patients while remimazolam 0.04 mg/kg with the trend of less respiratory adverse events was more optimal for elderly patients.

MOAA/S was commonly used for perioperative assessment of sedation level, especially for procedure sedation [13, 17]. Because ultrasound-guided nerve block could be completed in a short time, the aim of sedation depth in our study was set at MOAA/S score of 3–4. This was different from studies in endoscopy and bronchoscopy which required deep sedation (i.e., MOAA/S score of 1–2) to overcome relative strong stimulations [13, 18].

Remimazolam was usually given as a single fixed dose in most studies. A recent study simulating population pharmacodynamic with Markov mixed-effects model showed that 5 mg remimazolam with fentanyl provided superior sedation in comparison with dose of 4 or 6 mg [19]. Contrary to previous studies, we conducted a weight-based regimen of remimazolam administration, which was individualized for patient’s age. We proposed that adequate sedation could be reached with less dosage of remimazolam in elderly patients because of a decrement in physiological reservation (i.e., frail status). This was supported by Liu and colleague’s study which found that the median effective dose (ED50) and ED95 of remimazolam for anesthesia induction were both lower in patients aged above 70 years than those in younger patients [20]. Although body weight did not affect the systemic clearance of remimazolam, sedation level of remimazolam indeed increased positively with its dose. It was reported that in healthy volunteers, remimazolam dosed at 0.05 mg/kg only resulted in small reductions in MOAA/S scores (to 4) and BIS value (to 75) while doses of 0.075 mg/kg and above resulted in deeper sedation, as evidenced by MOAA/S scores of ≤ 2 and mean BIS value of 60–70 soon after dosing [21]. A weight-based regimen considering patients’ age for procedure sedation was more flexible and adhered to real-world clinical practice.

Our study showed that the success rate of procedure sedation was significantly increased in patients treated with remimazolam. To our knowledge, this was the first study to investigate the sedation efficacy of remimazolam during nerve block. In young patients, the success rate was positively associated with the increment of remimazolam dosage, i.e., from 43.3% in control group to 80.0% in high dose of remimazolam. In elderly patients, we also noticed that higher dose of remimazolam was associated with an increased trend of adverse effects such as respiratory depression and hypoxemia. Elderly patients also experienced longer duration of recovery time which was up to 15 min with remimazolam dosed at 0.06 mg/kg, which was consistent with previous study [19]. Clinical meaning of our data indicated that the observation time after procedure sedation should be extended in elderly patients even the elimination half-life of remimazolam was only several minutes.

Success rate of nerve block can be affected by many predisposing factors such as anxiety and sleep. Preoperative anxiety status and sleep quality was comparable among each group in the present study. Patients who received remimazolam had lower pain intensity during nerve block and higher score of self-reported satisfaction. It seemed that remimazolam may enhance the analgesic effect of opioids via synergistic effects [19].

Our study had some limitations. First, pharmacodynamic parameters of remimazolam with sufentanil were not examined. Second, dose of sufentanil was fixed in our present study, and further studies should be taken to search optimal combination regimen of remimazolam and sufentanil. Third, generalization of our result to other clinical scenario with severe pain procedure should be cautioned because these procedures may need more analgesics with opioids.

## Conclusion

Present study showed that supplementation of remimazolam to sufentanil improved the success rate of procedure sedation both in young and elderly patients during nerve block. Remimazolam 0.08 mg/kg provided the best sedation efficacy in young patients while remimazolam 0.04 mg/kg with the trend of less respiratory adverse events was more suitable for elderly patients.

## Supplementary Information

Below is the link to the electronic supplementary material.

Supplementary file1 (DOCX 34 KB)

## Data Availability

The dataset analysed in the current study is available from the corresponding author on reasonable request.

## References

[1] Chou R, Gordon DB, de Leon-Casasola OA, Rosenberg JM, Bickler S, Brennan T, Carter T, Cassidy CL, Chittenden EH, Degenhardt E, Griffith S, Manworren R, McCarberg B, Montgomery R, Murphy J, Perkal MF, Suresh S, Sluka K, Strassels S, Thirlby R, Viscusi E, Walco GA, Warner L, Weisman SJ, Wu CL (2016). Management of postoperative pain: a clinical practice guideline from the American Pain Society, the American Society of Regional Anesthesia and Pain Medicine, and the American Society of Anesthesiologists’ Committee on Regional Anesthesia, Executive Committee, and Administrative Council. J Pain.

[2] Koscielniak-Nielsen ZJ, Rotbøll-Nielsen P, Rassmussen H (2002). Patients’ experiences with multiple stimulation axillary block for fast-track ambulatory hand surgery. Acta Anaesthesiol Scand.

[3] Fuzier R, Lavidale M, Bataille B, Richez AS, Maguès JP (2010). Anxiety: an independent factor of axillary brachial plexus block failure?. Ann Fr Anesth Reanim.

[4] Jeng CL, Torrillo TM, Rosenblatt MA (2010). Complications of peripheral nerve blocks. Br J Anaesth.

[5] Borgeat A, Aguirre J (2009). Sedation and regional anesthesia. Curr Opin Anaesthesiol.

[6] Practice Guidelines for Moderate Procedural Sedation and Analgesia 2018: A Report by the American Society of Anesthesiologists Task Force on Moderate Procedural Sedation and Analgesia, the American Association of Oral and Maxillofacial Surgeons, American College of Radiology, American Dental Association, American Society of Dentist Anesthesiologists, and Society of Interventional Radiology. Anesthesiology. 2018;128(3):437–79. 10.1097/aln.0000000000002043 10.1097/ALN.0000000000002043 29334501

[7] Kubulus C, Schmitt K, Albert N, Raddatz A, Gräber S, Kessler P, Steinfeldt T, Standl T, Gottschalk A, Meissner W, Wirtz SP, Birnbaum J, Stork J, Volk T, Bomberg H (2016). Awake, sedated or anaesthetised for regional anaesthesia block placements?: A retrospective registry analysis of acute complications and patient satisfaction in adults. Eur J Anaesthesiol.

[8] Hinkelbein J, Lamperti M, Akeson J, Santos J, Costa J, De Robertis E, Longrois D, Novak-Jankovic V, Petrini F, Struys M, Veyckemans F, Fuchs-Buder T, Fitzgerald R (2018). European Society of Anaesthesiology and European Board of Anaesthesiology guidelines for procedural sedation and analgesia in adults. Eur J Anaesthesiol.

[9] Jones RD, Chan K, Roulson CJ, Brown AG, Smith ID, Mya GH (1993). Pharmacokinetics of flumazenil and midazolam. Br J Anaesth.

[10] Keam SJ (2020). Remimazolam: first approval. Drugs.

[11] Schüttler J, Eisenried A, Lerch M, Fechner J, Jeleazcov C, Ihmsen H (2020). Pharmacokinetics and pharmacodynamics of remimazolam (CNS 7056) after continuous infusion in healthy male volunteers: part I. Pharmacokinet Clin Pharmacodyn Anesthesiol.

[12] Zhang X, Li S, Liu J (2021). Efficacy and safety of remimazolam besylate versus propofol during hysteroscopy: single-centre randomized controlled trial. BMC Anesthesiol.

[13] Pastis NJ, Yarmus LB, Schippers F, Ostroff R, Chen A, Akulian J, Wahidi M, Shojaee S, Tanner NT, Callahan SP, Feldman G, Lorch DG, Ndukwu I, Pritchett MA, Silvestri GA, Investigators P (2019). Safety and efficacy of remimazolam compared with placebo and midazolam for moderate sedation during bronchoscopy. Chest.

[14] Marteau TM, Bekker H (1992). The development of a six-item short-form of the state scale of the Spielberger State-Trait Anxiety Inventory (STAI). Br J Clin Psychol.

[15] Andrzejowski J, Sleigh JW, Johnson IA, Sikiotis L (2000). The effect of intravenous epinephrine on the bispectral index and sedation. Anaesthesia.

[16] Chernik DA, Gillings D, Laine H, Hendler J, Silver JM, Davidson AB, Schwam EM, Siegel JL (1990). Validity and reliability of the observer’s assessment of alertness/sedation scale: study with intravenous midazolam. J Clin Psychopharmacol.

[17] Chen X, Xin D, Xu G, Zhao J, Lv Q (2022). The Efficacy and safety of remimazolam tosilate versus dexmedetomidine in outpatients undergoing flexible bronchoscopy: a prospective, randomized, blind, non-inferiority. Trial Front Pharmacol.

[18] Chen SH, Yuan TM, Zhang J, Bai H, Tian M, Pan CX, Bao HG, Jin XJ, Ji FH, Zhong TD, Wang Q, Lv JR, Wang S, Li YJ, Yu YH, Luo AL, Li XK, Min S, Li L, Zou XH, Huang YG (2021). Remimazolam tosilate in upper gastrointestinal endoscopy: a multicenter, randomized, non-inferiority, phase III trial. J Gastroenterol Hepatol.

[19] Zhou J, Curd L, Lohmer LRL, Delpratt N, Ossig J, Schippers F, Stoehr T, Schmith VD (2021). A population pharmacodynamic Markov mixed-effects model for determining remimazolam-induced sedation when co-administered with fentanyl in procedural sedation. Clin Transl Sci.

[20] Liu M, Sun Y, Zhou L, Feng K, Wang T, Feng X (2022). The median effective dose and bispectral index of remimazolam tosilate for anesthesia induction in elderly patients: an up-and-down sequential allocation trial. Clin Interv Aging.

[21] Antonik LJ, Goldwater DR, Kilpatrick GJ, Tilbrook GS, Borkett KM (2012). A placebo- and midazolam-controlled phase I single ascending-dose study evaluating the safety, pharmacokinetics, and pharmacodynamics of remimazolam (CNS 7056): part I. Safety, efficacy, and basic pharmacokinetics. Anesth Analg.

