# Supplementary material for: Sedation efficacy of different dose of remimazolam with sufentanil for nerve block in young and elderly patients: a randomized, controlled study
Source: J Anesth. 2022 Dec 3;37(2):177–85. doi: 10.1007/s00540-022-03142-8 (PMC10042748; doi:10.1007/s00540-022-03142-8)
Supplement: Supplementary file 1 — Supplementary file1 (DOCX 34 KB) [file 540_2022_3142_MOESM1_ESM.docx]

**Supplemental Table S1.** Pairwise comparation of outcomes in young patients.

|  | Group M vs. Group C | Group H vs. Group C | Group H vs. Group M |
| --- | --- | --- | --- |

|  | Estimated difference (95% CI) | *P* | Estimated difference (95% CI) | *P* | Estimated difference (95% CI) | *P* |
| --- | --- | --- | --- | --- | --- | --- |
| Primary outcome |  |  |  |  |  |  |
| Sedation success rate | RR=1.69 (1.07 to 2.69) | 0.018 | 1.85 (1.18 to 2.89) | 0.003 | 1.09 (0.83 to 1.44) | 0.542 |
| Secondary outcomes |  |  |  |  |  |  |
| Onset time (MOAAS≤4), min | MD= -1.0 (-2.0 to -1.0) | <0.001 | -1.0 (-2.0 to -1.0) | <0.001 | 0.0 (0.0 to 0.0) | 0.129 |
| Worst NRS of pain during the block, score | MD= -2 (-3 to -1) | <0.001 | -2 (-3 to -1) | <0.001 | 0 (-1 to 1) | 0.877 |
| Time consumption of the block, min | MD= 0.0 (-1.0 to 1.0) | 0.786 | 0.0 (-1.0 to 1.0) | 0.923 | 0 (-1.0 to 1.0) | 0.654 |
| Need rescue drugs | RR= 0.133 (0.03 to 0.53) | <0.001 | --- | --- | --- | --- |
| Recovery time, min | MD= 3.0 (1.0 to 4.0) | 0.004 | 4.0 (3.0 to 5.0) | <0.001 | 1.0 (1.0 to 3.0) | 0.124 |
| Self-reported satisfaction of patient, score | MD= 10 (5 to 20) | <0.001 | 20 (10 to 20) | <0.001 | 0 (0 to 0) | 0.064 |
| Self-reported satisfaction of anesthesiologist, score | MD=0 (0 to 5) | 0.458 | 0 (0 to 0) | 0.174 | 0 (0 to 0) | 0.319 |

NRS, numeric rating score

Estimated differences are presented as median difference (MD) and their 95% confidence interval (CI), relative risk (RR) and their 95% confidence interval (CI). P < 0.0167 is considered statistically significant after Bonferroni correction.

**Supplemental Table S2.** Pairwise comparation of outcomes in elderly patients.

|  | Group L vs. Group C | Group M vs. Group C | Group M vs. Group L |
| --- | --- | --- | --- |

|  | Estimated difference (95% CI) | *P* | Estimated difference (95% CI) | *P* | Estimated difference (95% CI) | *P* |
| --- | --- | --- | --- | --- | --- | --- |
| Primary outcome |  |  |  |  |  |  |
| Sedation success rate | RR= 1.58 (1.10 to 2.26) | 0.008 | 1.58 (1.10 to 2.26) | 0.008 | 1.00 (0.79 to 1.26) | >0.999 |
| Secondary outcomes |  |  |  |  |  |  |
| Onset time (MOAAS≤4), min | MD= -1.0 (-1.0 to 0) | <0.001 | -1.0 (-1.0 to -1.0) | <0.001 | 0 (0 to 0) | 0.133 |
| NRS pain score at the start of block, score | MD= -1 (-2 to 0) | 0.029 | -1 (-2 to 0) | 0.010 | 0 (0 to 0) | 0.548 |
| Time consumption of the block, min | MD= 0.0 (-1.0 to 1.0) | 0.978 | 0.0 (0.0 to 1.0) | 0.258 | 0.0 (0.0 to 1.0) | 0.370 |
| Need rescue drugs | RR= 0.27 (0.08 to 0.90) | 0.018 | --- | --- | --- | --- |
| Recovery time, min | MD= 1.0 (-1.0 to 3.0) | 0.228 | 5 (2 to 8) | <0.001 | 4 (2 to 7) | 0.001 |
| Self-reported satisfaction of patient, score | MD= 0 (0 to 10) | 0.003 | 10 (0 to 10) | <0.001 | 0 (0 to 0) | 0.413 |
| Self-reported satisfaction of anesthesiologist, score | MD= 0 (0 to 10) | 0.003 | 0 (0 to 0) | 0.152 | 0 (0 to 0) | 0.105 |

Estimated differences are presented as median difference (MD) and their 95% confidence interval (CI), relative risk (RR) and their 95% confidence interval (CI). P < 0.0167 is considered statistically significant after Bonferroni correction.

**Supplement Table S3.** Detailed information of regional block.

|  | Young patients | | | | Elderly patients | | | |
| --- | --- | --- | --- | --- | --- | --- | --- | --- |
| Types of regional block | Group C  (n= 30) | Group M  (n=30) | Group H  (n=30) | *P* | Group C  (n= 38) | Group L  (n=38) | Group M  (n=38) | *P* |
| **Trunk block** | **26 (86.7%)** | **29 (96.7%)** | **27(90.0%)** | **0.522** | **32** **(84.2%)** | **32** **(84.2%)** | **32** **(84.2%)** | **>0.999** |
| Rectus sheath block | 1 (3.3%) | 1 (3.3%) | 0 (0%) | >0.999 | 1 (2.6%) | 1 (2.6%) | 0 (0%) | >0.999 |
| Transverse abdominis plane block | 5 (16.7%) | 11 (36.7%) | 14 (46.7%) | 0.051 | 16 (42.1%) | 17 (44.7%) | 19 (2.6%) | 0.842 |
| Quadratus lumborum block | 9 (30.0%) | 8 (26.7%) | 3 (10.0%) | 0.149 | 2 (5.3%) | 3 (7.9%) | 3 (7.9%) | >0.999 |
| Erector spinal plane block | 6 (20.0%) | 2 (6.7%) | 5 (16.7%) | 0.413 | 3 (7.9%) | 3 (7.9%) | 4 (10.5%) | >0.999 |
| Thoracic paravertebral block | 5 (16.7%) | 7 (23.3%) | 5 (16.7%) | 0.839 | 10 (26.3%) | 8 (21.1%) | 6 (15.8%) | 0.577 |
| **Extremity block** | **4 (13.3%)** | **1 (3.3%)** | **3 (10.0%)** | **0.522** | **6** **(15.8%)** | **6** **(15.8%)** | **6** **(15.8%)** | **>0.999** |
| Axillary brachial plexus block | 3 (10.0%) | 0 (0%) | 0 (0%) | 0.104 | 2 (5.3%) | 0 (0%) | 1 (2.6%) | 0.772 |
| Supraclavicular brachial plexus block | 0 (0%) | 0 (0%) | 0 (0%) | --- | 0 (0%) | 1 (2.6%) | 1 (2.6%) | >0.999 |
| Femoral block | 1 (3.3%) | 1 (3.3%) | 1 (3.3%) | >0.999 | 2 (5.3%) | 3 (7.9%) | 2 (5.3%) | >0.999 |
| Sciatic block | 0 (0%) | 0 (0%) | 1 (3.3%) | >0.999 | 1 (2.6%) | 2 (5.3%) | 1 (2.6%) | >0.999 |
| Femoral & Sciatic block | 0 (0%) | 0 (0%) | 1 (3.3%) | >0.999 | 1 (2.6%) | 0(0%) | 1 (2.6%) | >0.999 |

**Supplement Table S4**. Exploratory analysis of satisfaction with category form.

|  | Young patients | | | | | Elderly patients | | | |
| --- | --- | --- | --- | --- | --- | --- | --- | --- | --- |
|  | Group C  (n= 30) | Group M  (n=30) | Group H  (n=30) | *P* | Group C  (n= 38) | | Group L  (n=38) | Group M  (n=38) | *P* |
| Patient satisfaction level, n |  |  |  | **<0.001** |  | |  |  | **<0.001** |
| Very satisfied | 13 (43.3%) | 28 (93.3%) | 27 (90.0%) |  | 25 (65.8%) | | 33 (86.8%) | 38 (100.0%) |  |
| Satisfied | 13 (43.3%) | 1 (3.3%) | 2 (6.7%) |  | 11 (28.9%) | | 3 (7.9%) | 0 (0%) |  |
| Neutral | 4 (13.3%) | 0 (0%) | 1 (3.3%) |  | 2 (5.3%) | | 2 (5.3%) | 0 (0%) |  |
| Dissatisfied | 0 (0%) | 1 (3.3%) | 0 (0%) |  | 0 (0%) | | 0 (0%) | 0 (0%) |  |
| Extremely dissatisfied | 0 (0%) | 0 (0%) | 0 (0%) |  | 0 (0%) | | 0 (0%) | 0 (0%) |  |
| Anesthesiologist satisfaction level, n |  |  |  | **0.006** |  | |  |  | **0.004** |
| Very satisfied | 18 (60.0%) | 25 (83.3%) | 24 (80.0%) |  | 26 (68.4%) | | 35 (92.1%) | 32 (84.2%) |  |
| Satisfied | 12 (40.0%) | 4 (13.3%) | 3 (10.0%) |  | 12 (31.6%) | | 2 (5.3%) | 4 (10.5%) |  |
| Neutral | 0 (0%) | 0 (0%) | 3 (10.0%) |  | 0 (0%) | | 1 (2.6%) | 0 (0%) |  |
| Dissatisfied | 0 (0%) | 1 (3.3%) | 0 (0%) |  | 0 (0%) | | 0 (0%) | 2 (5.3%) |  |
| Extremely dissatisfied | 0 (0%) | 0 (0%) | 0 (0%) |  | 0 (0%) | | 0 (0%) | 0 (0%) |  |
